# Advanced Visualization of Musculoskeletal Pathologies Using MV-Flow Ultrasound: A Case Series

**DOI:** 10.7759/cureus.73453

**Published:** 2024-11-11

**Authors:** Hye-Jin Y Clark, Clayton Walker, Eugene Y Roh

**Affiliations:** 1 Physical Medicine and Rehabilitation, Stanford University Medical Center, Redwood City, USA; 2 Sports Medicine, Physical Medicine and Rehabilitation, Department of Orthopedic Surgery, Stanford University Medical Center, Redwood City, USA; 3 Sports Medicine, Physical Medicine and Rehabilitation, Department of Orthopedic Surgery, Stanford University, Redwood City, USA

**Keywords:** diagnostic musculoskeletal ultrasound, microcirculation and inflammation, microvascular ultrasound, musculoskeletal imaging, mv flow ultrasound, sports related injuries, tendinopathy pain

## Abstract

The blood flow-detecting mode of ultrasound images can be beneficial for assessing the degree of inflammation among various musculoskeletal conditions and their recovery. Power Doppler (PD) ultrasound is typically used for blood flow, but its limitations in detecting low-velocity blood flow hinder comprehensive assessment. The microvascular flow tool (MV-Flow) from the Samsung V7 and RS85 ultrasound systems (Samsung Co., Seoul, South Korea), offers advanced visualization of microcirculatory and slow-flow connections that PD and Color Doppler (CD) cannot detect. This case series highlights the novel application of MV-Flow in diagnosing sports medicine-related conditions, specifically tendinopathy, demonstrating its utility even when magnetic resonance imaging (MRI) and conventional ultrasound fail to reveal abnormalities.

## Introduction

The evaluation of musculoskeletal conditions has traditionally relied on imaging modalities such as magnetic resonance imaging (MRI) and ultrasound [1]. While these imaging techniques provide critical insights into the structural and pathological aspects of musculoskeletal disorders, understanding the underlying microcirculation is equally essential, as it significantly influences muscle function, plasticity, and rehabilitation success [2]. Power Doppler (PD) has been extensively utilized in musculoskeletal ultrasound, particularly for evaluating structures to detect inflammation and soft tissue neoplasms. While PD is preferred over color Doppler (CD) due to its increased sensitivity to low-velocity blood flow, it is still challenging to evaluate microvasculature in certain body regions [3,4]. The microvascular flow (MV-Flow) tool included with the V7 and RS85 ultrasound system (Samsung Co., Seoul, South Korea) visualizes microcirculatory and slow-flow connections that conventional techniques cannot identify [5]. Conventional PD displays the magnitude of color flow instead of the Doppler frequency signal. Thus, the PD feature filters motion artifacts, which leads to the loss of low-flow components. Consequently, conventional methods cannot detect blood flow with a velocity below the constant (<200 Hz).

The MV-Flow tool employs a filter system different from the one used by Doppler imaging; the former reduces artifacts from random motion while preserving the detection of directional motion from flowing blood. These advanced signal processing and adaptive filtering techniques result in a greater sensitivity to lower velocity blood flow evaluation. Hence, the MV-Flow imaging’s improved ability to detect small vessels reduces the need for further computed tomography (CT) and MRI imaging; both of these modalities are relatively expensive, involve a higher risk of radiation with CT, and can be potentially nephrotoxic if using intravenous contrast agents [6].

To our knowledge, there have not yet been any studies using MV-Flow to diagnose musculoskeletal conditions. In the present case series, the MV-Flow tool was used to diagnose and treat various sports medicine-related symptoms and conditions. While the MV-Flow tool is often used in the evaluation of fetal microvascular and hepatic flow, this novel function can be expanded to diagnose tendinosis and injuries relevant to sports medicine.

## Case presentation

Materials and methods

We reviewed ultrasound cases from 2023 to 2024 performed by a single provider who was board-certified in sports medicine and musculoskeletal ultrasound. The diagnostic ultrasound was performed using either the Samsung V7 or RS85 machines. The linear array transducer with a frequency range of 2-14 MHz (LA2-14A) was used to locate the area of diseased tissue. Once identified, the MV-Flow tool was activated and used to identify regions of hyperemia. Minimal probe pressure was used to prevent occluding blood flow and the MV-Flow sensitivity was adjusted to optimize image quality. Next, the CD tool was activated, and using the same technique, the same area was scanned looking for hyperemia. Screenshots were captured using each mode for comparison. All the patients had an MRI of the affected body part done either before or shortly after the diagnostic ultrasound, which was used to confirm the diagnosis and serves as a control in this case series. Formal approval by the Institutional Review Board was not required due to this being a limited retrospective case series.

Case reports

Case 1

**Figure 1:**
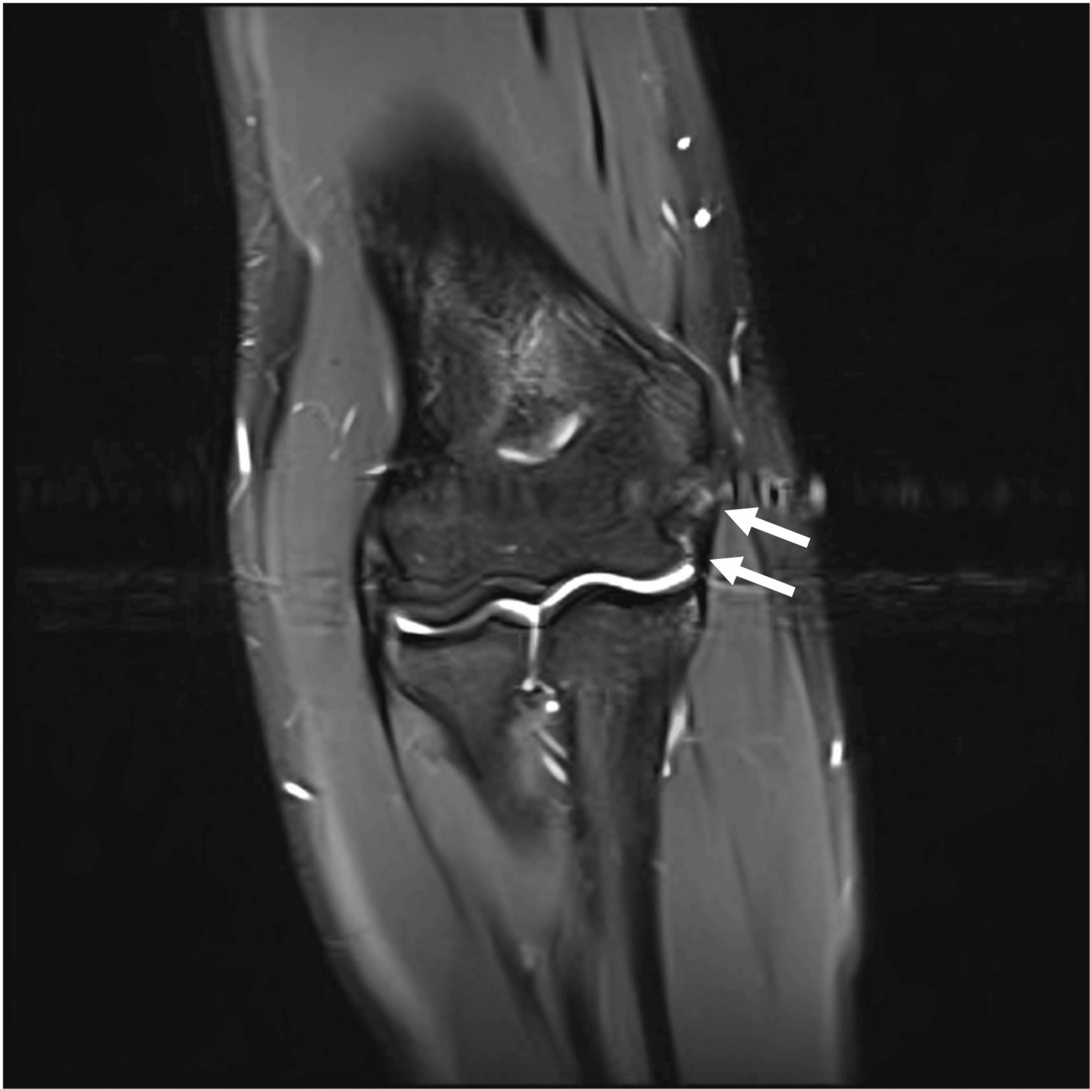
Coronal STIR MRI view of a 17-year-old man showing UCL partial tear and mild osseous irregularity at the attachment of the UCL, which is mildly thickened anteriorly with minimal edema. Short T2 inversion recovery (STIR); magnetic resonance imaging (MRI); arrows: ulnar collateral ligament (UCL).

**Figure 2:**
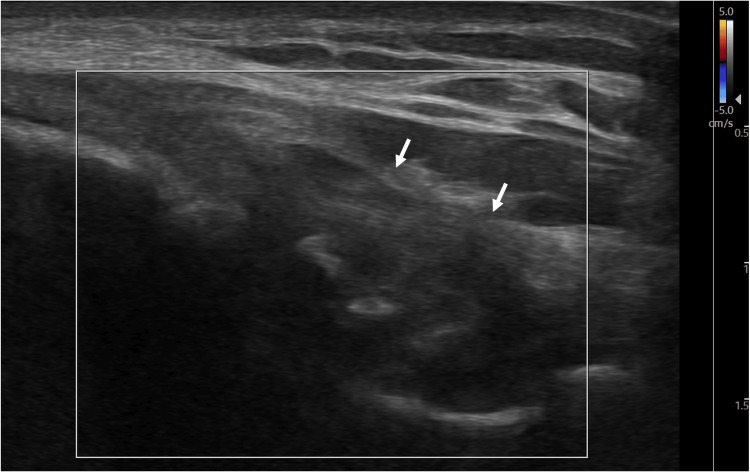
Long-axis view of the UCL with the color Doppler tool not visualizing any hyperemia. Arrows: Ulnar collateral ligament (UCL).

**Figure 3:**
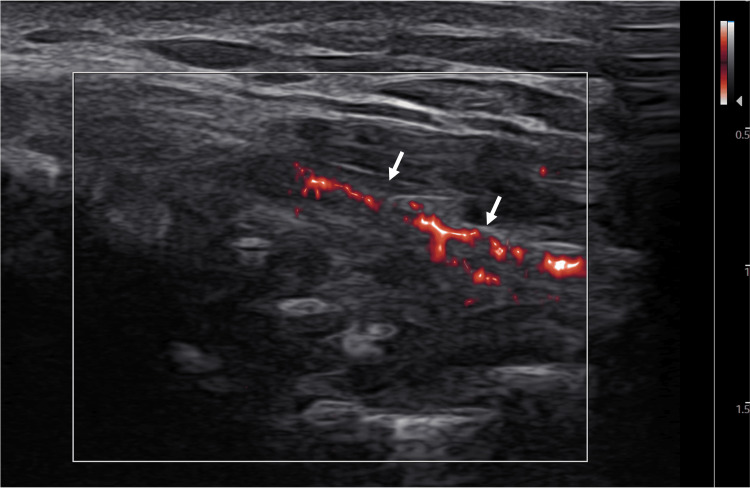
Long-axis view of UCL with MV-Flow tool showing hyperemia within the ligament. Arrows: Ulnar collateral ligament (UCL).

A 17-year-old male high school baseball catcher presented to the sports medicine clinic with right medial elbow pain while throwing. While the pain was partially resolved with physical therapy, the pain recurred about six months earlier while playing baseball to the point where he had to take a month off from the sport. A physical exam demonstrated mild tenderness to palpation to the medial epicondyle. MRI of the right elbow demonstrated subacute to chronic high-grade partial tearing of the proximal ulnar collateral ligament (UCL) (Figure 1). Plasma-rich platelet (PRP) injection under ultrasound guidance was recommended. During the treatment procedure, point-of-care ultrasound (POCUS) was used was used for assessment and needle guidance. While the conventional CD function did not show any hyperemia (Figure 2), the MV-Flow tool depicted diffuse hyperemia in the proximal portion of the UCL (Figure 3).

Case 2

**Figure 4:**
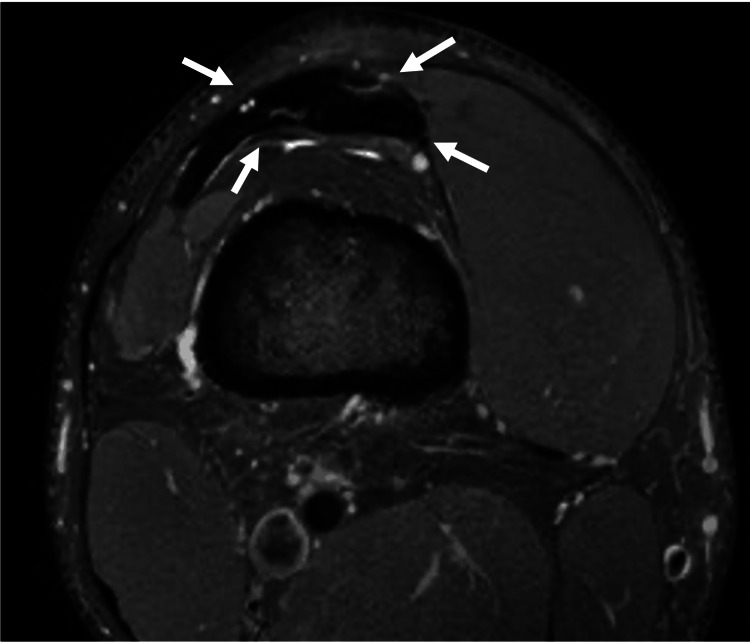
Mild quadriceps tendinosis in a 21-year-old man. Axial proton density fat-suppressed MRI of the knee showing mild distal quadriceps tendinopathy. Arrows: Quadriceps tendon.

**Figure 5:**
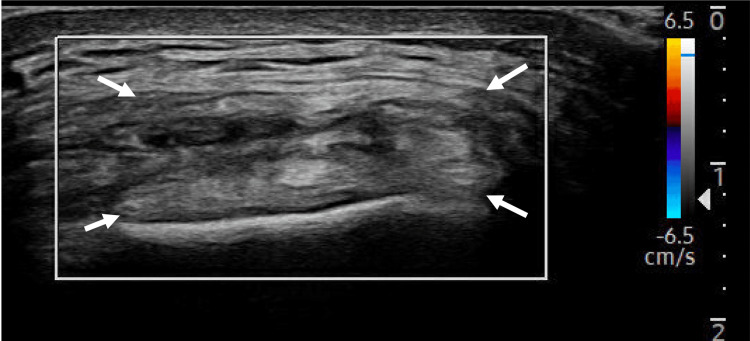
Short-axis view of the distal quadriceps tendon with color Doppler showing no hyperemia within the tendon. Arrows: Quadriceps tendon.

**Figure 6:**
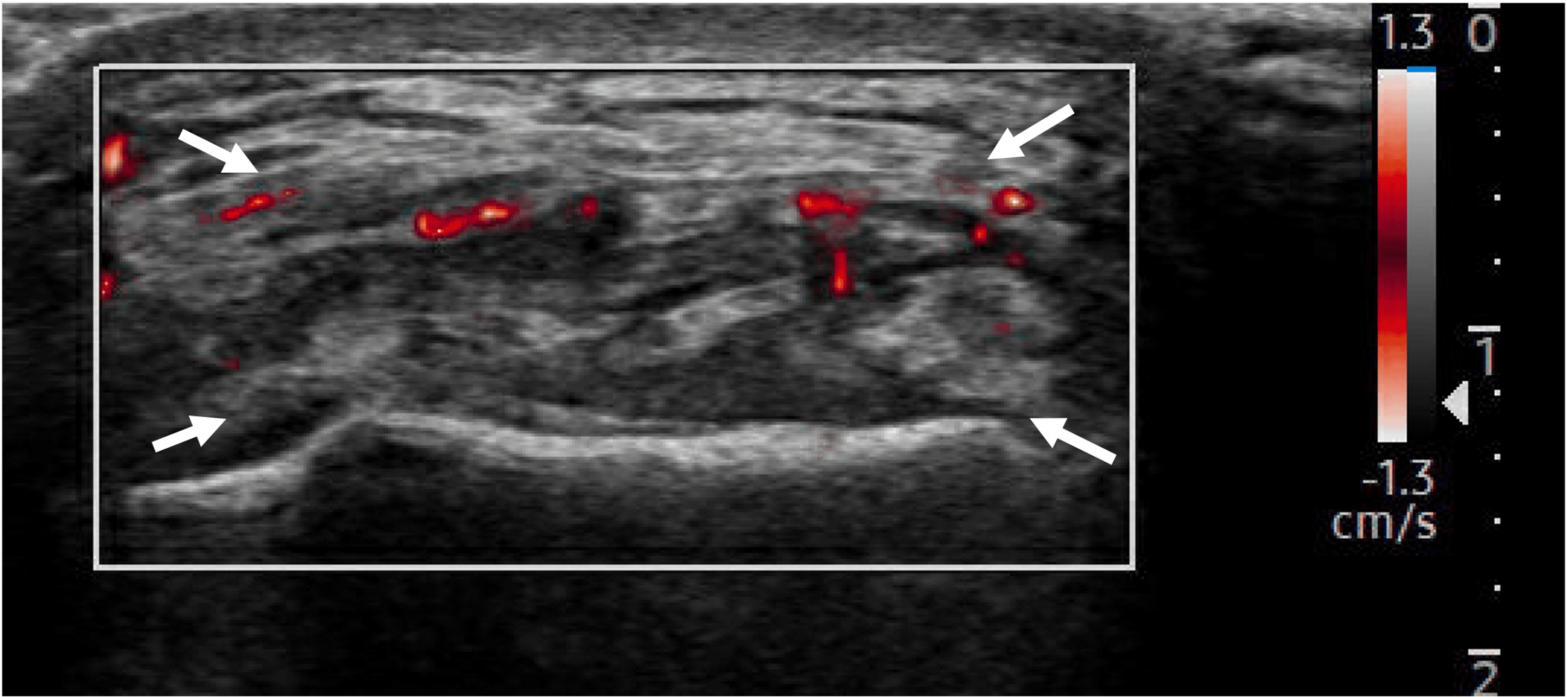
Short-axis view of the distal quadriceps tendon with MV-Flow tool showing hyperemia within the tendon. Arrows: Quadriceps tendon.

A 21-year-old male collegiate athlete presented with several months of right knee discomfort. The pain was localized to the distal quadriceps tendon and was worse with squatting and lunging. On examination, the patient had tenderness to palpation of the distal quadriceps tendon. An MRI revealed mild distal quadriceps tendinopathy and superior patellar enthesopathy (Figure 4). Extracorporeal shock wave therapy was recommended. Prior to starting treatment, limited POCUS was performed to serve as a baseline, which would later be used to compare to post-treatment images to assess the success of the extracorporeal shock wave therapy. The MV-Flow tool demonstrated hyperemia at the distal portion of the quadriceps tendon that was not visualized using CD (Figures 5-6).

Case 3

**Figure 7:**
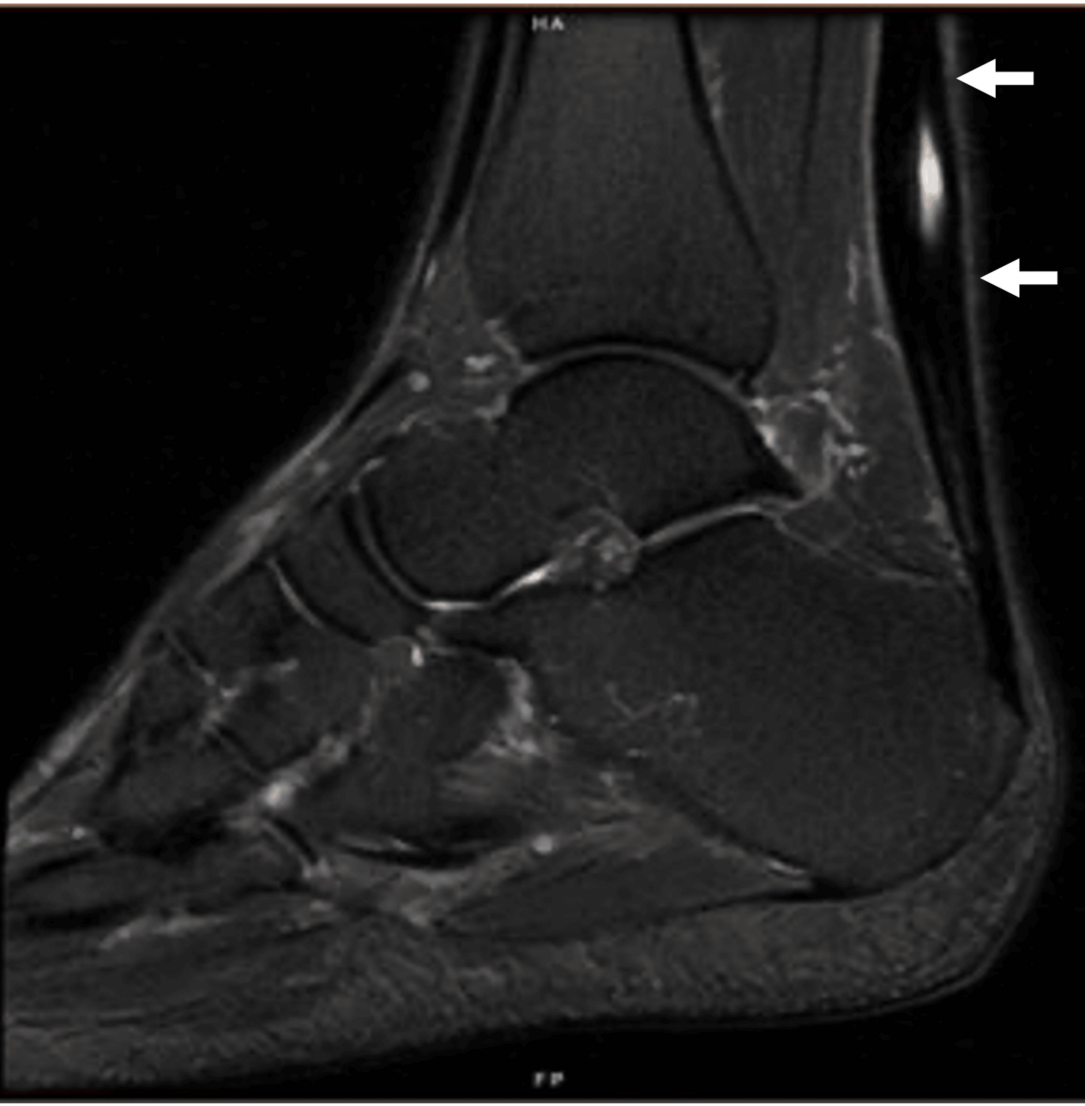
Sagittal fat-suppressed T2 MRI of the ankle showing mild Achilles tendinosis with an interstitial tear. Arrows: Achilles tendon.

**Figure 8:**
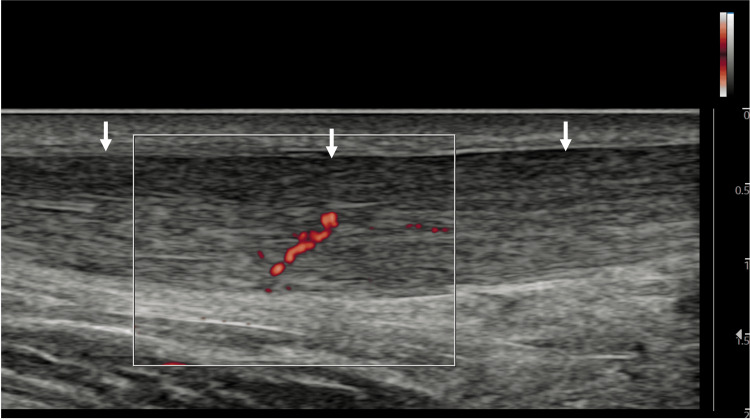
Achilles tendinosis in a 57-year-old man. Long-axis view of Achilles tendon with MV-Flow showing hyperemia within the tendon. Arrows: Achilles tendon.

**Figure 9:**
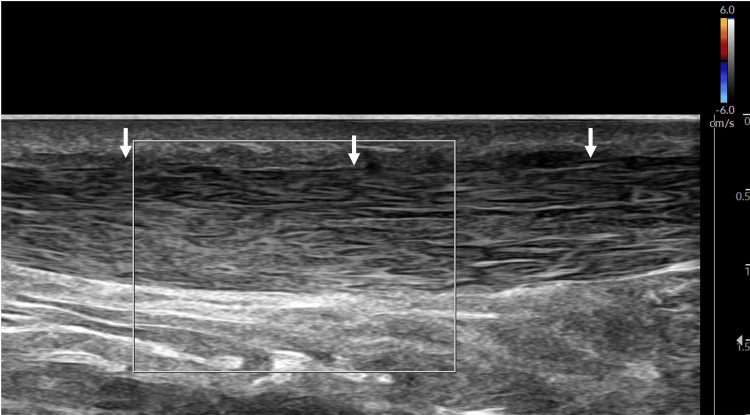
Long-axis view of the Achilles tendon with the color Doppler tool showing no hyperemia within the tendon. Arrows: Achilles tendon.

A 57-year-old male avid recreational golf player presented with right heel pain that began four years ago. The pain was localized to the lateral Achilles tendon, radiated up to the calf, and was exacerbated with walking. On physical exam, there was notable thickening of the Achilles tendon that was tender to palpation. MRI of the right ankle confirmed chronic Achilles tendinosis with an intra-substance tear (Figure 7). The patient then underwent an ultrasound-guided percutaneous needle tenotomy, during which MV-Flow was obtained to precisely visualize the diseased tissue with hyperemia to guide the focus of the treatment. At the follow-up visit, while a limited POCUS showed that the previously visualized cystic lesion on the MRI had resolved, the patient reported improved but residual pain. MV-Flow demonstrated a small hyperemic lesion under the location of pain in the Achilles tendon (Figure 8) that was not detectable with CD (Figure 9). 

Case 4

**Figure 10:**
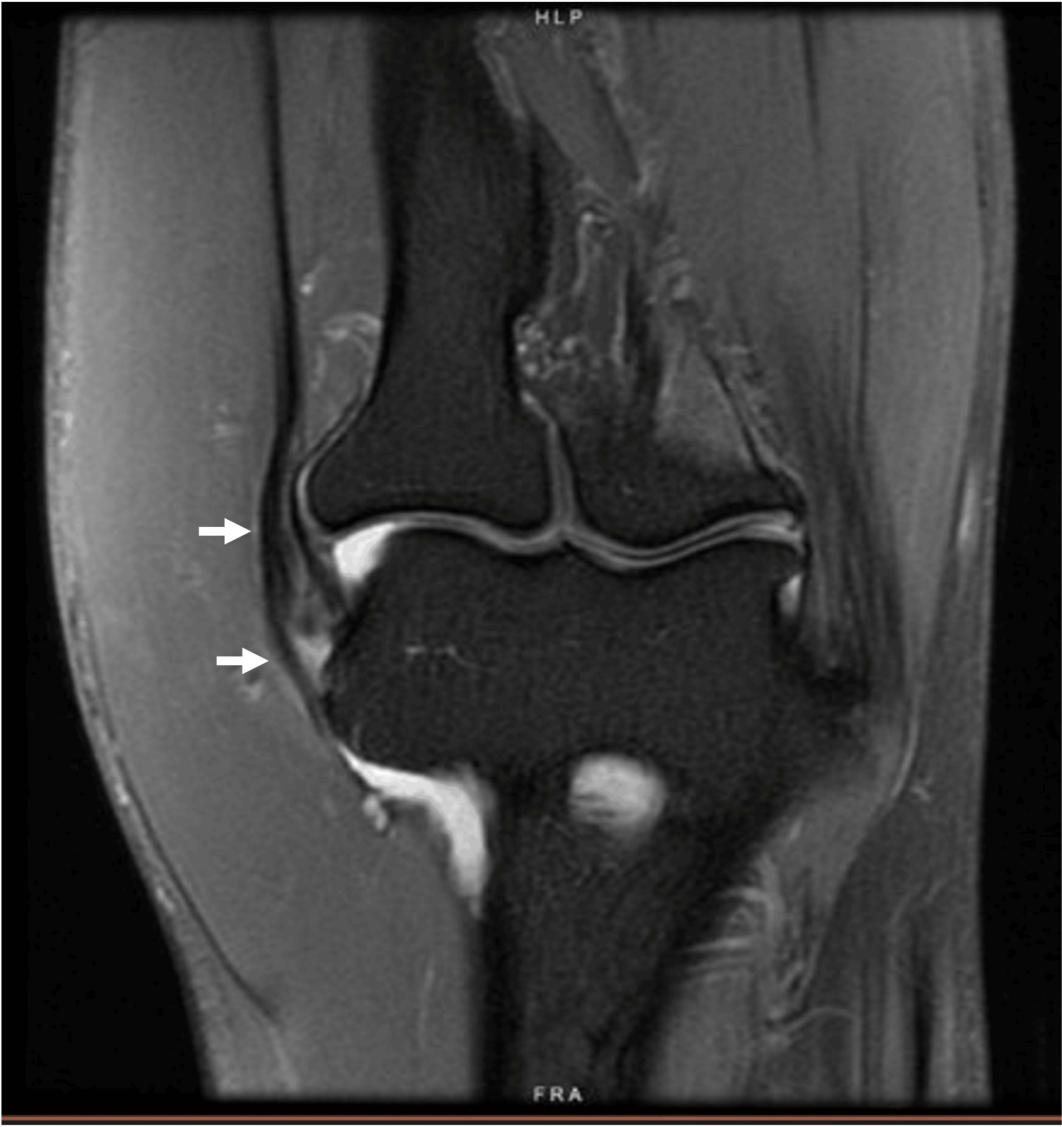
Tendinosis of the common extensor tendon at the lateral epicondyle attachment in a 53-year-old man. Coronal proton density fat-suppressed MRI of the elbow showing a low-grade partial thickness tear of the proximal common extensor tendon at the lateral epicondyle attachment. Arrows: Common extensor tendon.

**Figure 11:**
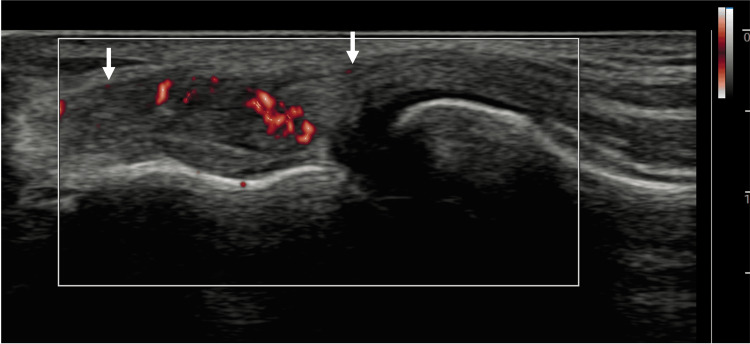
Long-axis view of the common extensor tendon with MV-Flow showing moderate hyperemia within the tendon. Arrows: Common extensor tendon.

**Figure 12:**
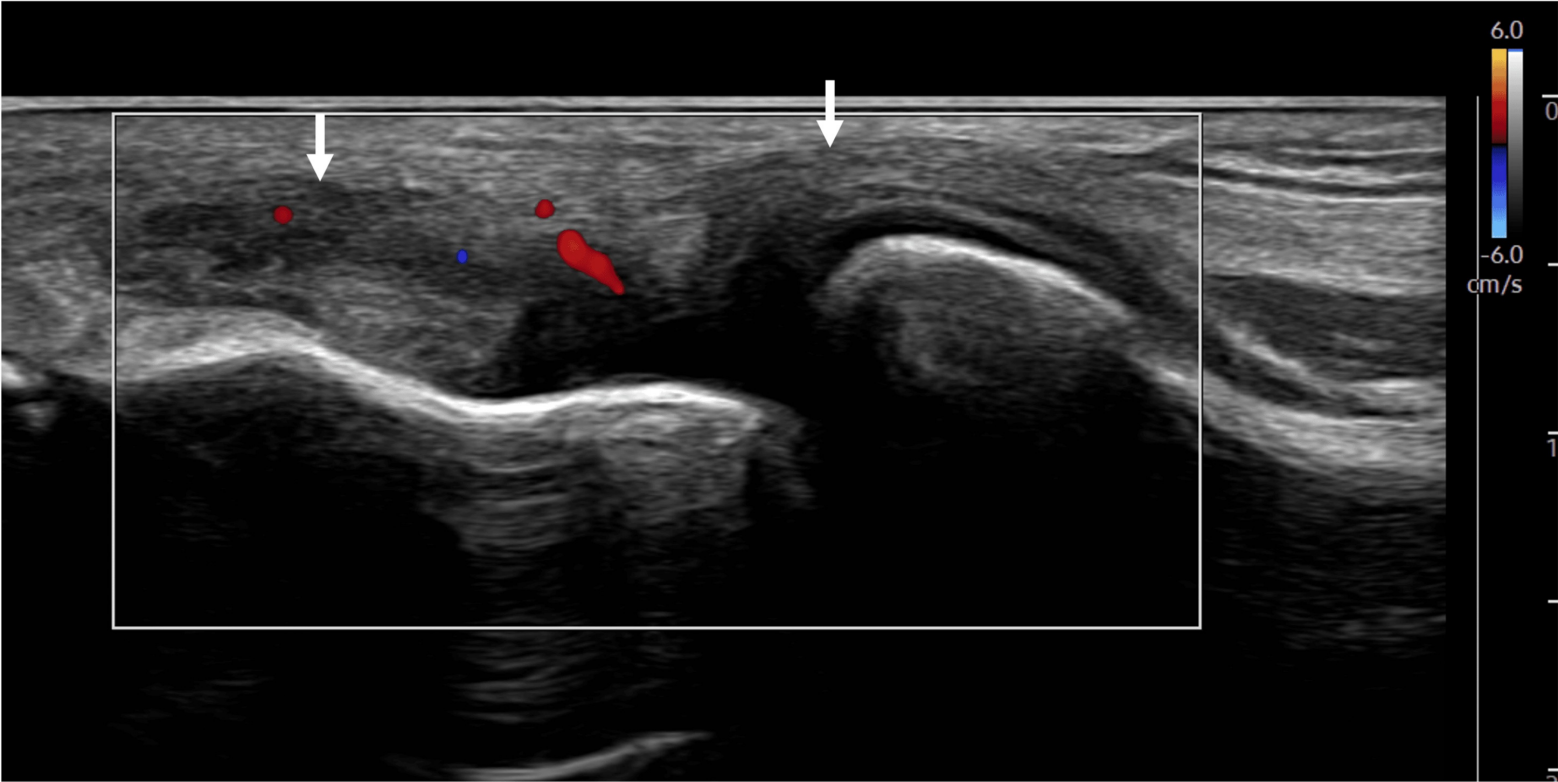
Long-axis view of the common extensor tendon with the color Doppler tool showing mild hyperemia within the tendon. Arrows: Common extensor tendon.

A 53-year-old male presented with right elbow pain for the past 1.5 years. His pain was localized to the lateral epicondyle region with intermittent radiation down the elbow. On exam, the patient had discomfort with resisted wrist flexion and resisted finger extension. MRI revealed a low-grade partial-thickness tear of the proximal common extensor tendon at the lateral epicondyle attachment (Figure 10). A limited POCUS demonstrated an anechoic lesion in the common extensor tendon. MV-Flow demonstrated diffuse hyperemia throughout this region, while the conventional CD only showed focal hyperemia, which impacted how the severity of the injury was graded (Figures 11-12).

Case 5

**Figure 13:**
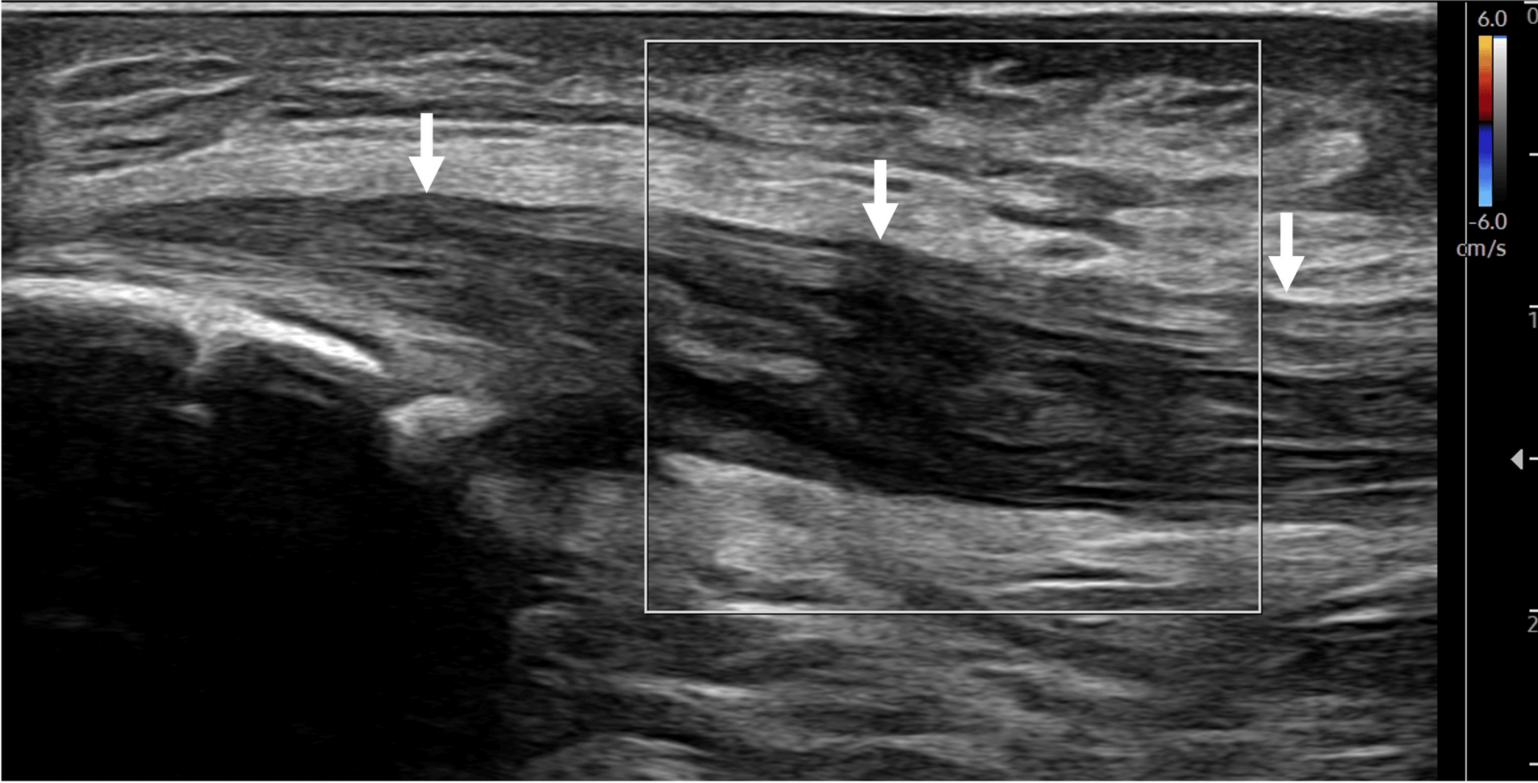
Tendinosis of the proximal patellar tendon in a 22-year-old man. Long-axis view of the patellar tendon with Color Doppler tool showing no hyperemia within the tendon Arrows: Patellar tendon.

**Figure 14:**
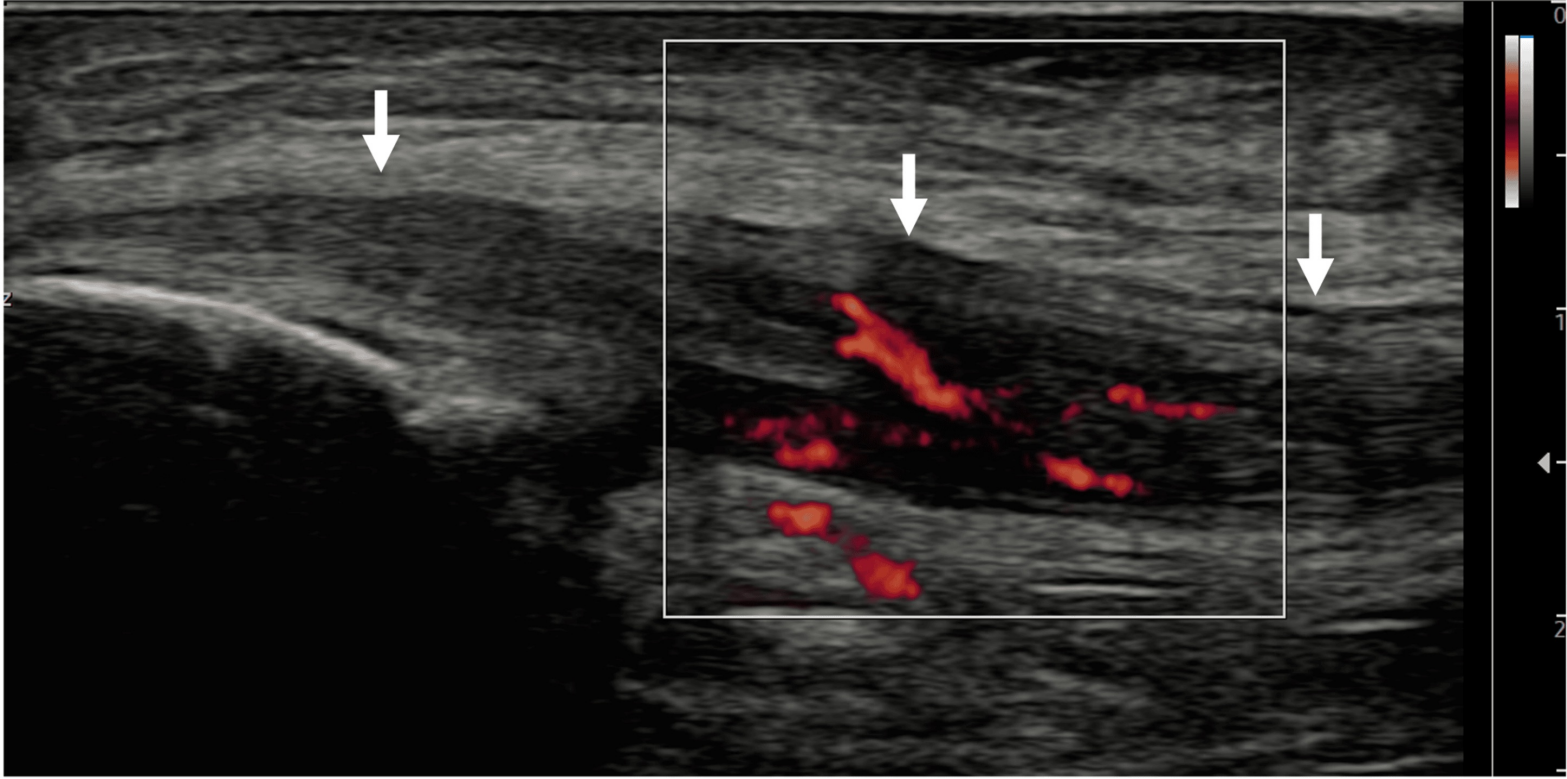
Long-axis view of the patellar tendon with MV-Flow tool showing hyperemia within the tendon. Arrows: Patellar tendon. NOTE: MRI images for this patient were not available for publication.

A 22-year-old male elite-level football player with a history of anterior cruciate ligament (ACL) reconstruction with bone-patellar tendon-bone graft techniques one year ago was referred for persistent left anterior knee pain. The physical exam was notable for left knee crepitus with active extension and tenderness to palpation but no ligamentous laxity. MRI showed appropriate postoperative changes from the ACL reconstruction with mild patellar tendinosis. On further evaluation with POCUS of the left patellar tendon, the CD did not show hyperemia (Figure 13) while the MV-Flow mode detected a significant area of hyperemia throughout the proximal portion of the tendon (Figure 14). MV-Flow was then used to successfully guide a PRP injection into the area of diseased tissue. 

## Discussion

The findings of this case series underscore the enhanced capabilities of the MV-Flow tool in assessing microcirculation in musculoskeletal conditions, particularly in tendinopathy in sports-related injuries. Tendinopathy is reported to account for 30-50% of all overuse injuries and has a prevalence of 15% in elite athletes [7-9]. Ultrasound is a reliable, non-invasive, and cost-effective tool to support the clinical diagnosis of tendinopathy [10]. Yet, traditional imaging tools within the ultrasound, including CD and PD, often fail to detect low-velocity blood flow, which is crucial for diagnosing and treating conditions with subtle microvascular abnormalities. This limitation can lead to incomplete assessments and potentially missed early diagnoses; however, by removing the tissue motion artifacts, while maintaining a high frame rate, the MV-Flow tool allows the assessment of blood microvascular perfusion at a higher resolution than the Doppler techniques [11].

Enhanced sensitivity to microcirculation

The MV-Flow tool's advanced signal processing and adaptive filtering techniques enable it to detect lower-velocity blood flow more effectively than conventional CD and PD. By offering a distinct advantage of visualizing the microvasculature of lesions, MV-Flow enables quantitative analysis of tissue blood supply at various stages and from different lesions [12]. This was evident across all cases presented, where MV-Flow revealed areas of hyperemia and small vessel flow that were not visible with CD. For instance, in Case 1, MV-Flow identified diffuse hyperemia in the UCL of a baseball player, facilitating targeted treatment with a PRP injection. Similarly, in Case 3, the tool detected an area of hyperemia in the Achilles tendon that conventional CD failed to reveal. Even when common extensor tendon hyperemia was detected by the conventional CD as seen in Case 4, the MV-Flow tool showed more diffuse hyperemia, which improved the accuracy of grading tendinosis severity.

Diagnostic precision and clinical implications

The ability of MV-Flow to detect microvascular changes where other modalities cannot have significant clinical implications. For patients with sports injuries, timely and accurate diagnosis is critical for appropriate intervention, recovery, and return to play. In previous studies, 60% of elite athletes with patellar tendinopathy showed structural tendon changes and neovascularization in painful areas on CD ultrasound but not in pain-free normal tendons [13]. The MV-Flow tool provides a non-invasive, radiation-free, and cost-effective alternative to MRI and CT scans, which reduces the need for these studies while adding enhanced precision in detecting the area of neovascularization that the conventional CD cannot. In Case 5, for example, MV-Flow was instrumental in identifying inflammation in the patellar tendon of a football player and was used to accurately guide the PRP injection to the area of diseased tissue.

Limitations and future directions

While this case series highlights the potential of MV-Flow in diagnosing tendinopathies and ligament sprains, further studies are needed to validate its efficacy across a broader range of musculoskeletal conditions. Additionally, future studies using larger patient populations and multiple providers would improve the generalizability of the findings.

Expanding applications

Previously, the MV-Flow tool has been used to monitor fetal growth restriction and for hepatic imaging after chemotherapy in liver cancer patients, and most recently, has shown good diagnostic efficacy for ovarian adnexal masses [14-16]. Its application in sports medicine represents a novel and promising expansion. To date, there are no reports of MV-Flow being used to diagnose tendinopathy and subsequent ultrasound-guided treatments. The case series demonstrates that MV-Flow can provide critical diagnostic information at the bedside that would otherwise require an evaluation with an MRI. These techniques can be applied to diagnose patients accurately and quickly or to monitor a patient's response to various treatments, including physical therapy, extracorporeal shock wave therapy (ESWT), and PRP injections.

Expanding on the role of MV-Flow in sports medicine could represent a significant advancement in the field, especially given the increasing demand for objective tools to monitor treatment responses. Patients frequently request measurable ways to assess their progress following PRP or ESWT [17-18]. The ability to visualize and quantify microvascular changes using MV-Flow could potentially offer an objective measure of treatment efficacy, providing real-time insights into the healing process. 

This is particularly important because most studies evaluating the effectiveness of PRP have primarily relied on subjective pain scores [19-20]. Microvascular imaging could revolutionize assessing treatment responses, offering a more comprehensive approach combining subjective and objective data. Consequently, MV-Flow may improve diagnostic accuracy and offer a novel method for tracking rehabilitation success in musculoskeletal conditions. This could lead to enhanced treatment strategies and more personalized care for patients undergoing therapies such as PRP and ESWT.

## Conclusions

The MV-Flow provides greater diagnostic accuracy through visualization of low-velocity blood flow in the microvascular of the examined structure, offering superior sensitivity compared to traditional Doppler methods. This case series is the first to demonstrate its utility in diagnosing sports-related injuries, enabling more precise and effective treatments. Additionally, this technology could serve as a valuable tool for evaluating tendon conditions pre- and post-treatment, potentially enhancing the assessment of recovery and treatment efficacy. Future research should focus on larger, controlled studies to further establish the clinical utility in diagnosing different musculoskeletal injuries.
